# An Essay on Croup

**Published:** 1856-09

**Authors:** 


### ECLECTIC DEPARTMENT,

### AND SPIRIT OF THE MEDICAL PERIODICAL PRESS.

---

### *An Essay on Croup.* By S. M. Bemiss, M. D.

(Concluded.)

*Prognosis.*—The prognosis of croup is, in the large majority of cases, unfavorable. The presence and nature of complications, the relaxation or increased severity of the symptoms detailed, determine its character. Patients of croup may die from suffocation by mechanical closure of the air tubes, produced by the thickness of the false membrane and swelling of the tissues beneath; from suffocation by spasmodic closure of the glottis; from exhaustion by defibrinization of the blood (Rokitansky); from fibrinous concretions of the heart (B. W. Richardson); from prolonged incomplete asphyxia, and its poisoning influence upon the blood; and from the attendant complications.

*Treatment.*—In looking over the pages of different authors upon the treat ment of croup, the reader is struck with the great number of remedies proposed for its relief. But, as has been well remarked by Vauthier, "in therapeutics numerous remedies more often indicate poverty than riches," and the necrological reports fully demonstrate that fertility of resources in reference to this disease, has sadly failed to deprive it of its fatality. It is even a matter for solemn and responsible inquiry, whether remedial agents may not in some instances have served to lengthen the mortuary lists. With my present views of the pathology of croup, I think the physician should enter upon its treatment with a constant reference to the laws connected with the formation and disposal of its exudation. As a disease of the mucous membrane, apart from the exudation, "it is often a trivial one," but it is this concrete deposit which gives the malady its fatal character; consequently the limitation, and removal of this formation are of vital consequence to the successful issue of every case. Before undertaking any remedial procedure, especially such as may compromise the strength of his patient, the physician should keep in view the tendencies of this concretion when left to nature. Dr. Bennett says, "when the exudation has coagulated it constitutes a foreign body which can only be removed by its becoming organized or by its dying." We have seen that organization of this membrane never occurs; "dying" and purulent decomposition *always* occur, if the life of the patient is preserved sufficiently long for these processes to be effected. Hence the practitioner may find cases where the safety of his patient is better insured by trusting to this slow but certain natural process for his relief. (Ware.)

It is important also to recollect that the exudative process is not necessarily a condition of active inflammation, local or general; we should therefore carefully avoid exhausting our patients by combating an element which may not exist. With these views I regard the following to be the indications of cure:

1. To limit the exudation and promote its expulsion.
2. To treat complications when present.
3. To sustain the strength of the patient.
4. To form an artificial opening for the air when the occlusion of its natural passages renders it expedient.

To avoid repetition I shall consider the several remedies in connection with the indication which their leading properties seem best to fulfill, and then pursue them through any other indications to which they may apply.

1. *To limit the exudation, and promote its expulsion.—Bloodletting.*—No remedy of equal power has been so lavishly and indiscriminately resorted to, in croup, as bloodletting. It has been practiced from the first announcement of the disease to the deep coma of collapse, as I have witnessed, with an equally unsparing hand; and often, as I conceive, with fatal effect. I believe, however, that bloodletting may sometimes prove advantageous, if resorted to at the very onset of the disease, and in a sthenic condition of the system. I would admit it, then, not for the purpose of reducing inflammatory symptoms, so much as to detract from the mass of the circulation, and by derivation reverse the action of the vessels pouring forth the deposit. But if full dilatation of these vessels has occurred, and the membrane is established over a considerable surface, bleeding should not be practiced. It is even problematical whether it has the good effects imputed to it above. Copeland mentions having seen the disease attack young children "soon after having lost much blood in the treatment of other diseases." Valliex quotes a case from Bretonneau, in which a "very abundant local bleeding was followed by a prompt extension of the disease." I should prefer venesection to local bleeding, as being more under control and probably less liable to agitate the patient; but with my present feelings of distrust in its beneficial effects, I should assume the responsibility with the utmost reluctance, and only after the most careful investigation of symptoms. In croup, complicated with pneumonia, it is clearly contra-indicated.

*Emetics.*—I regard emetics as among our most potent means in answering the present indications. In the early stages they should be given with a view to effect revulsion in the circulation, and to solicit emulgence of the salivary and muciparous glands, and thus relieve the local congestion. The specific emetics are therefore to be preferred, and of these I think the end best attained by ipecac, to which, if a full effect be desired, a small portion of tartrate of antimony may be added. Later in the disease, when the only use of remedies of this class is to free the fauces of tenacious mucus, and to encourage the expulsion of the membrane by the concussion of the act of vomiting, the indications are best answered by the irritant emetics, such as the sulphates of zinc, alumina and copper, whose effects are prompt, and terminate with the act of emesis. These remedies, especially the sulphate of alumina and potash, have at times enjoyed the reputation of specifics for croup. In addition to their emetic properties, their astringent and alterant effects upon the throat no doubt contribute somewhat to their curative influence. It is best, whichever of these remedies be employed, that it should be always administered in doses sufficiently large to insure prompt emesis. The superiority of emetic over nauseating doses of medicine in the treatment of this disease, is clearly proven by the valuable observations of M. Valleix. "Of thirty-one patients who were treated with energetic vomits, twenty-six expelled false membranes in the efforts of vomiting, and of this number fifteen recovered. Of twenty-two patients with whom vomiting was employed in a timid manner, and as secondary medication, only two rejected false membranes, one recovered, the remaining twenty *rejected no membrane, and all died.**

The persistent use of antimony in nauseating doses is, I think, seldom or never productive of benefit, and often occasions the most alarming and pernicious prostration of strength.

The frequency with which emetics should be repeated, is a point of as much importance as the selection of the agent. If we fail to give them as often as indicated, we do not secure their full benefits; if we administer them too frequently, we hazard the strength and proper support of our patient. No certain rules can be laid down upon this point. When intelligent nurses are in attendance, they should be instructed to administer an emetic whenever the throat seems very much clogged with tenacious mucus, or when suffocation is imminent. Their use is to be deprecated unless some such indication calls for it; and we ought especially to avoid giving them, in the latter stages of the disease, so soon after nourishment has been taken, as to cause its rejection.

*Calomel.*—It has been very generally admitted that calomel seems to exert some control over exudative processes, though in what means this influence is exerted, is a point difficult of explanation. My experience leads me to regard its action in croup with favor, and, except in very ataxic conditions of the system, I always resort to its use. I generally employ it in cathartic doses at first, and afterward as an alterative, and continue it, at suitable intervals, until amelioration of symptoms occurs, in cases of recovery, and in fatal cases, until collapse supervenes. I sometimes prescribe it alone, or, if a strong pulse or dry skin exist, combined with small portions of ipecac; or Dover's powder, if active catharsis is apprehended.

*Topical Applications.*—The attention of the medical world has recently been much invoked to this mode of treatment. Very few doubt the beneficial effects to be derived from topical medication in affections of the respiratory ways, if feasible, but many deny the practicability of making such applications. Among the latter class is Mr. Erichsen, who states, "that there is no reliable evidence that the sponge probang has ever been passed through and beyond the vocal cords." The limits of this essay will not permit me to discuss this important question further than to remark that it is difficult to reject the mass of testimony disproving the assertion of Dr. Erichsen. Yet I am far from willing to accept all the instances of reported introduction of the sponge within the larynx, as true. I imagine that the operation is rarely performed, and so difficult of accomplishment, that in making local

---

* Guide du Medicin Practicien.

applications in croup, the physician is not justifiable in regarding the penetration of the larynx as the *sine qua non* of beneficial influence. I do not doubt that the most practicable method of introducing the sponge within the larynx, is that described by Sir Charles Bell. After stating the strength of his solution and the curve of his wire probang, he thus describes his procedure: "With the finger of my left hand I pressed down the tongue, and stretched the forefinger over the epiglottis, then directing the wire along my finger, I removed the point of the finger from the glottis, and introduced the pad of lint into the opening, and pressed it with my finger." A similar method has been practiced by Dr. Palmer, of this city. We may readily realize the difficulty of this manipulation upon the diminutive organs, and amid the struggles of the child, although the object was only to reach the lips of the glottis.

I think, however, that benefit may and does accrue from this species of medication, without penetrating the larynx; and I attribute those beneficial influences to its detersive effect upon the throat, to the local influence of the solution upon the parts it reaches, and to the frequent dislodgement of membrane it occasions by the succussion and vomiting attending the application. That the local action of nitrate of silver upon exudating surfaces is beneficial, is abundantly proved by M. Empis, who witnessed its modifying influence upon the cutaneous exudation of diptheritis.

Some cases are recorded where topical applications alone seemed to have jugulated the disease, and, from analogy, I am persuaded that such instances would frequently occur, if the application could be had recourse to early, before the exudation has appeared to any extent upon other surfaces than the pharyngeal.

Dr. Eben Watson's experiments* led him to conclude that the more intense the local inflammation was, the more feeble the solution should be. I am unable to decide upon the propriety of this conclusion so far as regards its effects upon the local blood stasis and the infiltrated membrane, but consider an important result from its use to be its obtunding influence upon the increased nervous susceptibility of these parts; and for the purpose of producing this effect, think the stronger solutions are indicated. Guersent considers it best to use a very concentrated solution, and apply it but once a day. I am satisfied it is the best practice to use a strong solutions and apply it from one to three times in twenty-four hours.

2. *To Treat the Complications.*—The laryngismus is the most common complication of croup; in truth it is rarely absent, and to whatsoever cause it may be due, its control is at all times an important indication of treatment. The best remedies for this purpose are probably opium and hyoscyamus, but as the former sometimes acts irregularly on the constitution of children, preference should be given to the latter. This narcotic, administered in doses of from half a grain to a grain of the extract every two or four hours, had, in some cases under my charge, a most happy influence in calming the spasmodic action of the laryngeal muscles. Dr. Parrish, of Philadelphia, speaks very highly of the anodyne effects of injections of assafœtida and laudanum. Whenever there is found decided tendency to periodicity in the recurrence of paroxysms, aside from that induced by sleep, quinine is indicated. In a

* Braithwaite's Retrospect, No. xvi.

very large majority of cases of croup, I am satisfied that its effects, if not beneficial, would at least be innocuous; so that if a doubt exists as to the propriety of its use, it may still be employed, without fear of evil consequences. Its good effects in an uncomplicated laryngismus are generally recognized by the profession.

Pneumonia, bronchitis, and the exanthemathous diseases are common complications of croup. They should be met by their usual treatment modified according to the debilitated condition of the patient. Guersent considers bronchitis as not an unfavorable complication, since the profuse secretion to which it gives rise favors the solution of the false membrane.

3. *To sustain the strength of the patients.*—The importance of this indication is obvious to the physician who bears in mind the fact that separation of the membrane and cure of the patient will spontaneously result if life be sustained a requisite period; consequently, if the usual tendency of the disease to death by suffocation cannot be averted we should at least exercise care that the patient does not perish from exhaustion. An adequate amount of simple nourishment should be allowed throughout the disease, and the first indication of failing vital powers met by wine whey and weak toddy. Instead of producing arterial excitement, these stimulants, especially when given warm, have often been followed by favorable diaphoresis and a sensible mitigation of the expectoration.

The most remarkable recovery from croup that I ever witnessed, seemed to date from the moment the patient was again placed to his mother's breast, and the free use of weak toddy enjoined.

*Summary of Treatment.*—When a physician is called for the first time to see a case of croup, and by inspection of the fauces, and a careful grouping together of the symptoms, establishes the diagnosis, a point of great importance is to ascertain the stage of development of the disease. If he is so fortunate to see his patient during the stage of invasion or even immediately after the symptoms of confirmation have announced themselves, he may often hope to cut short the attack by the institution of vigorous treatment. The means best calculated to secure this end are the topical application of nitrate of silver, and the administration of an energetic vomit of ipecac and tartrate of antimony, followed by cathartic doses of calomel and the adjuvants yet to be mentioned. There are, I think, but few, if any, conditions of the disease, except that of collapse, where these remedies might not with propriety constitute the initiatory treatment. The first steps of treatment are, however, very important, since they frequently exercise an influence, either favorable or unfavorable, which lasts until the termination of the case. Unfortunately, circumstonces often surround the physician to embarrass his decisions; such as the urgency of the patient's symptoms, and the distress of surrounding relatives, and their importunities for more active interference. Without reference to these, however, he should trust to the calm guidance of his own judgment, and adapt his treatment to the exigencies of the case. If the attack be sporadic, the system in a sthenic state, and the symptoms have proceeded with rapid march, he may with less hesitation resort to active medication; but if this is not followed by immediate and obvious benefit, it should be at once abandoned. It is far better to trust to the spontaneous decomposition of the deposit, than to persist in lowering measures, when they are found to detract from the patient's strength without arresting the progress of the disease.

If, on the contrary, the case has been in existence for a number of days, if the preceding catarrh has been long, and the symptoms of invasion slow in exhibiting themselves, and the progressive aggravation is either not manifest or not strongly marked, we may be justified in trusting to nature for the removal of the membrane, and direct our attention only to the general state of the system, to the modification of the spasmodic element in the disease by appropriate anodynes, and to the general surrounding influences. I regard an attention to this last one of the essentials to a successful result. The child should be kept free from all unnecessary agitation; every excitement, emotional or physical, adds to the difficulty of the respiration, and the increased poisoning of the blood; consequently the presence of strangers around the bed should be avoided, and all interruptions prohibited unless demanded by the urgency of the symptoms. It is probable that the remarkable success of Dr. Budd's treatment with the vapor bath, was in part due to the fact that his patients were kept in curtained beds free from interruption.

External remedies, such as blisters, baths, narcotic and emollient cataplasms have long been in use in the treatment of croup. I have never seen any evidence of good results from blisters, and reject them from the list of remedies. Tobacco has been the most common narcotic used endermically; it certainly controls spasm, but so often induces alarming prostration, that I never countenance its employment. I have attributed good effects, in some instances, to frequent inunction of the neck with soap liniment and laudanum. Warm poultices are soothing, but their weight is an inconvenience. The objections to baths, are the agitation and alternations of temperature to which they subject the patient. I regard, however, the influence of temperature and moisture as a most valuable adjuvant in the treatment of croup. The powerful influence of cold in diminishing the circulation of parts to which it is applied is well known. Dr. Bennett, in his "Pathology and Treatment of Exudation," gives as an indication of prevention and cure, "topical application of cold and astringents which stimulate the capillaries to contraction." A number of cases are on record where the cure of the disease has been attributed to the external employment of cold. Dr. Gore, of Bloomfield, Kentucky, has furnished me the notes of two cases of recovery, in which he ascribes benefit to the constant application of ice cloths to the throat. I think the application of cold should be restricted to the early stages of the disease, and its indication limited to the prevention of exudation. If used in this stage, I should advise a low degree of temperature as better calculated to affect the deeper seated capillaries, and the better to secure its anæsthetic influence upon the laryngeal muscles. If the membrane be already formed to a considerable extent, the cold can of course do nothing to favor its removal, may even counteract the natural process of purulent metamorphosis, and should then be exchanged for external warmth. Jurine long since recommended vapor baths, and Dr. Budd has recently employed them with the most remarkable success. The plan of Dr. Budd is simply to place the patient in a curtained bed, and, to prevent fright from the novelty of its position, its mother with it; then to place upon the foot of the bed a large vessel of boiling water, into which a heated brick is occasionally plunged; the curtains are then to be drawn around the bed, and the child kept in a state of quietude, the temperature being maintained at from 75° to 80°. It seems to me that almost every curative indication of confirmed croup is answered by this admirable arrangement. The laryngismus is subdued, the breathing of the child quieted, while the warmth and moisture hasten the solution of the deposit, invite the circulation to the surface, and promote diaphoresis.

In addition to the remedies already mentioned, I have been in the habit of administering carbonate of soda in the latter stages of croup, upon the basis of an opinion long since advanced, that it favors the solution of the false membrane. Senega, squills, and tolu are also indicated in the latter stages.

In concluding this brief notice of the medical treatment of croup, I take the opportunity of quoting Valleix's instructions relative to the personal management of the patient:

"1. Lay the patient in such a position that he will have his head a little more elevated than his body.

"2. Remove from the chest and throat all clothes that embarrass the action of the parts.

"3. Preserve the air of the chamber at a warm temperature—guard the patient from currents of air.

"4. Give him drinks frequently, to remove the dryness of the throat and introduce a large quantity of fluid in the system."*

4. *To form an artificial opening for the air, when the occlusion of its natural passages renders it expedient.*—The expediency of bronchotomy in croup has been variously estimated. While some have gone so far as to contend that the physician who neglected its performance was chargeable with the death of his patient,† others have discountenanced the operation, as not increasing the chances of recovery, and consequently not worthy of the support of the profession.‡ Undoubtedly, the old adage will apply here, "*in medias res tutissimus ibis.*" The statistics of the operation show that it is worthy of countenance, since it affords the patient a "plank of safety" when all others are removed. At the same time, the great number of failures should check any enthusiasm, tending to induce too much confidence in the operation, to the exclusion of sufficient medical means.

In France, where this operation is resorted to much earlier and under more favorable circumstances than elsewhere, statistics show a proportion of about one in five to be successful. Velpeau records 140 cases and 28 recoveries. Jousset 219 cases and 40 cures. Mr. Lawrence (Lond. Lancet for Dec., 1855), 216 cases and 47 cures. According to the observations of Guersent, Weber, and Ware, about 45 would recover out of 216 cases of confirmed croup, left without operation, but were we to take the same number of cases at the period when bronchotomy is usually resorted to, and trust them to medical treatment alone, I am satisfied the mortality would largely exceed that of those submitted to operation. I think, therefore, that preservation of human life is, in the aggregate, subserved by bronchotomy, although

* Guide du Medicin Practicien.

† M. Trousseau se contente de dire que le Médecin qui ne pratique pas la Bronchotomie est oupable de la morte du malade.—*Jousset.*

‡ Experience has shown that the operation has failed in the great majority of cases; and it is obvious that with our present knowlege of the nature of the disease, we can scarcely hope for good from its performance.—*Stokes.*

cases occur where recovery would have taken place without the operation, and others again where fatal results follow in consequence of its performance.

The question being decided as to the propriety of the operation, it is an important point to know *when* it is expedient. This can only be determined by the gravity of the case and the discernment of the physician. Guersent says, "when the child is menaced with asphyxia, and the treatment fails to give relief, a fatal termination is imminent. We should not stop to consider the situation or extent of the small membranes; the child will die in five or six hours or sooner, and tracheotomy, however doubtful the result, is rigorously indicated. If the false membranes have only attained the larynx, the case is favorable; but delay lessens the prospect of success; in croup, as in strangulated hernia, we should always, without precipitating the operation, have the probable necessity of performing it in our mind."* The objection of parents is one of the most common obstacles to the performance of bronchotomy in croup. The physician can, however, conscientiously assure them that the operation is not itself dangerous, and that it is comparatively painless on account of the anæsthesia which the poisoned blood induces.—(Meigs.)

When bronchotomy is decided upon, it remains for the physician to choose between tracheotomy and laryngotomy. The former operation is the more difficult, but when performed with the requisite precautions, probably more successful, since it forms the opening with more certainty, between the point of obstruction and the lungs. Laryngotomy is more simple, more readily and rapidly performed, and if the operator could assure himself that the membrane extended but a little way into the trachea, should always be selected. It is not unlikely that too much stress has been laid upon the necessity of penetrating the trachea below the membrane. Dr. Craig† has published a very interesting case of successful tracheotomy, in which the membrane reached an inch below the lowest point of the incision. M. Saloy‡ has related a case of successful laryngotomy where the membrane also extended below the incision. Whichever operation is selected, a free incision should be made into the tracheal canal, for the strong current of air favors the ejectment of the membrane. It also enables the physician to facilitate the removal by the forceps or sponge; or if he possess the professional devotion, and self-forgetfulness of Roux, Saloy, and Erichsen, he may place his lips to the wound, and, by a strong inspiration, suck into his own mouth the blood, mucus and membranous debris, which frequently prevent respiration after the opening is completed.

Bronchotomy does not seem to have been often practiced in the West, in the treatment of croup. Dr. Gross, of this city, during his residence in Cincinnati, performed it upon a child nine months old, a patient of Drs. Wood and Rives, of that city. The disease had existed upwards of a week, producing great exhaustion. Some improvement followed, but it was only transient; in a short time the symptoms were as bad as ever, and death ensued in less than twenty hours after the operation.

Dr. J. B. Flint has operated once: his patient was moribund and the operation unsuccessful. Drs. Eve and Haskins, of Tennessee, have both operated, the former in two cases, the latter in one; their patients were all in extremis,

---

* Braithwaite's Retrospect, No. XIV.

† London Lancet for July, 1853.

‡ Archives Generales Mai 1849.

and all died. Dr. Asbury Evans, of Covington, has operated once. His operation, although performed under disadvantageous circumstances, gave the most flattering promises of success. Dr. E. remained with his patient, a boy of eight years, for twelve hours succeeding the operation; he then left him "calm, free from pain, breathing without difficulty—had slept easily," shortly after his departure, the imprudent mother, contrary to express injunctions, "raised him up to give him a drink—the tube slipped out, the patient himself tried to replace it, but not succeeding in penetrating the trachea, perished before assistance could arrive."

Other instances have undoubtedly occurred in the West, in which the operation has been performed for croup, but want of time has prevented me from obtaining authentic information respecting them.

In conclusion, the writer does not flatter himself that he has thrown any new lights upon either the nature or treatment of this disease, but if he succeeds in attracting the attention of the medical profession in the West, to the importance of the subject, and to the necessity of gathering new facts for the elucidation of its obscure pathology, and the improvement of its treatment, so as to abate its mortality, his object will be achieved.—*Louisville Review*.

